# Pneumatized Nasal Septum Extending to the Left Frontal Sinus "Septo-Frontal Cell": A Case Report

**DOI:** 10.7759/cureus.48831

**Published:** 2023-11-15

**Authors:** Rani Hammoud, Fatima Emam, Adham Aljariri, Emad Al Duhirat, Mansour Al Sulaiti, Hamad Al Saey, Ahmed Shaikh

**Affiliations:** 1 Otolaryngology - Head and Neck Surgery, Hamad Medical Corporation, Doha, QAT; 2 Radiology, Hamad Medical Corporation, Doha, QAT

**Keywords:** computed tomography, frontal sinus, pneumatized nasal septum, septoplasty, anterior nasal septum

## Abstract

Multiple anatomical variations in the nasal cavity are well-described in the literature. We describe a rare case of pneumatization of the frontal sinus in the nasal septum that we term "Septo-Frontal Cell". To the best of our knowledge, this pattern of nasal septum pneumatization has not been described in the literature before. We have discussed the clinical and radiological findings and management of this patient.

## Introduction

Identifying and understanding the complex three-dimensional structures of the nasal cavity and paranasal sinuses (PNS) along with their frequent anatomical variations is crucial for otolaryngologists in the planning of sino-nasal, nasal, and other skull-base surgeries [1]. Multiple anatomical variations have been described in the literature, some of which are commonly seen in most of the population while others can be quite rare. The frequency of these structural variations differs among ethnic groups [2]. We are describing a case report of a middle-aged Middle Eastern man whose PNS computed tomography (CT) revealed an extension of the left frontal sinus into a pneumatized bony nasal septum, undergoing septoplasty for a deviated nasal septum.

## Case presentation

A 39-year-old Middle Eastern male patient, not known to have any comorbidities, presented to our ENT clinic with a complaint of long-standing, left-sided nasal obstruction, associated with recurrent episodes of headache. The patient had no history of rhinorrhea, facial pain, reduced smell sensation, recurrent sneezing, nasal itchiness, nasal trauma, or previous nasal surgeries.

The general physical appearance revealed a healthy-looking adult with no craniofacial abnormality and the nasal examination showed severe right-sided columellar dislocation and left-sided septal deviation with bilateral moderately enlarged inferior turbinate more on the right side. Internal nasal examination using a zero-degree endoscope showed a left nasal septal spur touching the left inferior turbinate; otherwise, the remaining nasal examination was within normal limits.

**Figure 1:**
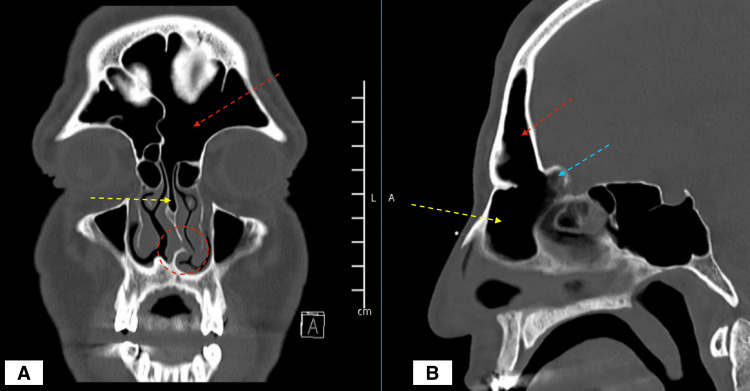
CT scan of the paranasal sinuses; A: coronal cut; B: sagittal cut red arrow: the left frontal sinus; yellow arrow: pneumatized anterior nasal septum; blue arrow: pneumatised crista galli; red circle: left nasal septal spur

A CT scan of paranasal sinuses showed a left nasal septal spur contacting the left inferior turbinate with a pneumatized bony nasal septum continuous with the left frontal sinus and a pneumatized crista galli (Figure 1).

**Figure 2:**
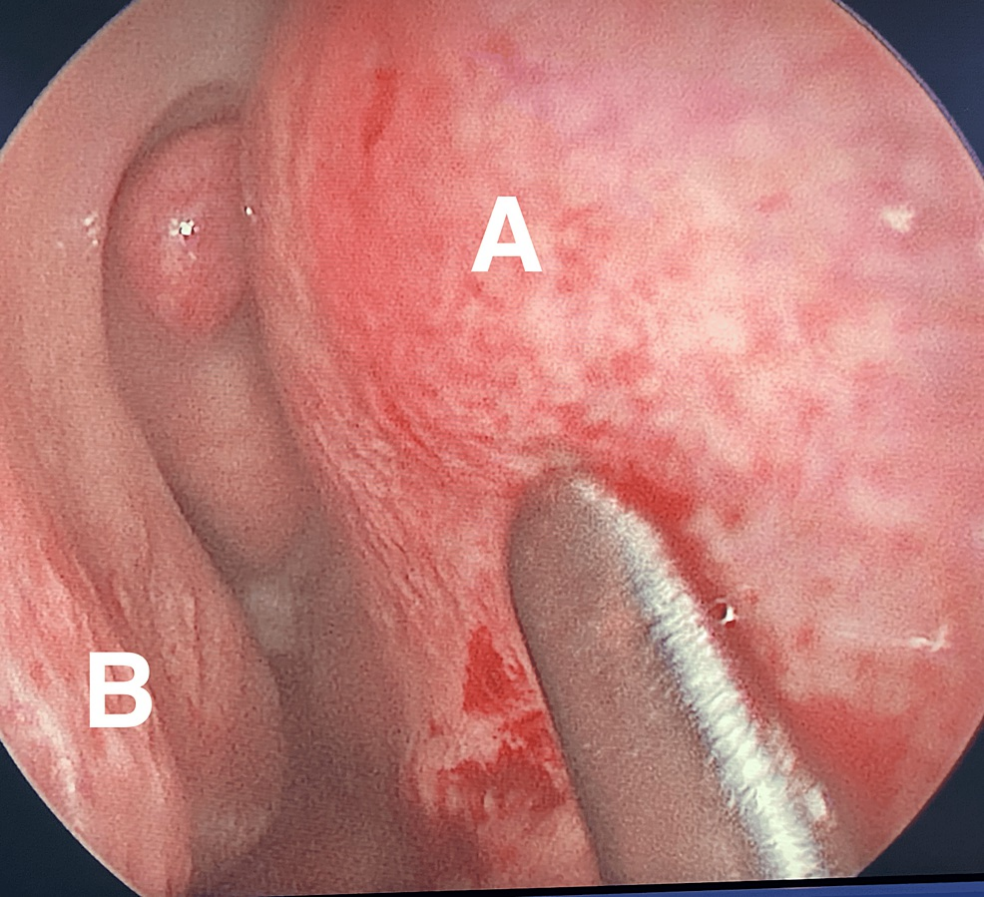
Nasal endoscopic showing the right-side nasal cavity A: pneumatized part of the nasal septum; B: right inferior turbinates

The patient underwent septoplasty with bilateral submucosal diathermy of the inferior turbinate, intra-operative findings showed an S-shaped nasal septum with columellar dislocation to the right side, severe left-sided septal spur, and deviated maxillary crest to the left. The pneumatized part of the nasal septum was not obstructing the airway and hence was not corrected (Figure 2). The patient recovered well after the surgery, with marked improvement in nasal breathing.

## Discussion

Anatomical sino-nasal variations, such as the deviated nasal septum, the presence of agger nasi, Haller and Onodi air cells, concha bullosa, and paradoxical middle turbinate, are commonly seen among the population [1,3]. Pneumatization can occur in any part of the sino-nasal area, with the pneumatization of the middle turbinate termed concha bullosa being the most frequently encountered [4] while the inferior nasal concha, crista galli, clivus, and perpendicular plate of ethmoid are less commonly pneumatized [5]. The frontal sinus anatomy can be either simple or complex. The international frontal sinus anatomy classification (IFAC) was developed to improve the surgeon's understanding of the variation of the frontal sinus anatomy and drainage. This classification described the anterior cells (agger nasi (AN), supra agger, and the super agger frontal cells), the posterior cells (supra bulla, supra bulla frontal, and supraorbital ethmoid cells), and the septal pneumatization of the frontal sinus that is referred as the frontal septal cell [6].

The preoperative CT scan sinus in our patient shows in addition to the nasal spur, which is causing the patient’s symptoms, an over-pneumatized frontal sinus along with bilateral agger nasi, a pneumatized crista galli, and a frontal septal cell. These findings are not uncommon, agger nasi cells are seen anterior to or just above the insertion of the middle turbinate in the lateral wall, and their reported prevalence ranges from 10% to 90% [7] while the prevalence of the frontal septal sinus is estimated to range between 8.3% to 30% and the prevalence of previously reported pneumatization of the crista galli ranges from 2.4% to 13% [8,9]. This case is unique in its pneumatized anterior nasal septum that is continuous with the above frontal septal cell, left frontal sinus, and pneumatized crista Galli (Figure 1).

**Figure 3:**
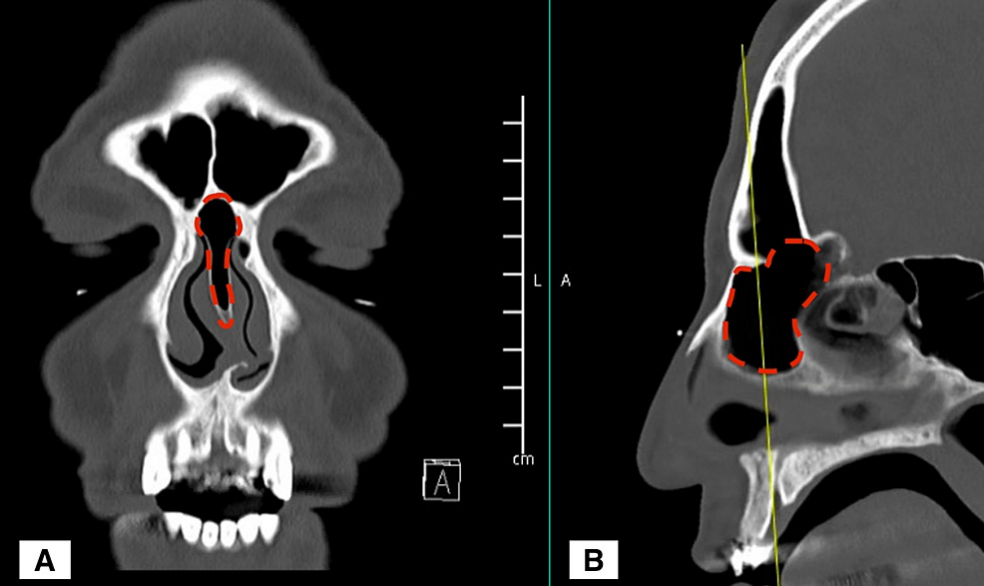
CT scan of the paranasal sinus; A: coronal cut; B: sagittal cut The area marked in red represents the Septo-Frontal Cell.

The pneumatized nasal septum, when present, is usually related to the sphenoid sinus [8] and is not taken into consideration when preparing for a septoplasty, given its posterosuperior location. However, we observed in our patient, an over-pneumatized frontal sinus with a frontal cell that is extending to the anterior nasal septum; we termed it a "Septo-Frontal Cell" (Figure 3).

Given the patient's complaint of nasal obstruction secondary to the deviated nasal septum, the patient opted for nasal septoplasty. A tailored approach for the septoplasty was crucial to avoid a potential anticipated complication, as the inadvertent opening of this cell can lead to either frontal sinus fistula in the septum or recurrent frontal sinusitis. Therefore, intraoperatively, we carefully corrected the nasal septal deviation by removing the left nasal septal spur while avoiding over-resection of the nasal septum; the Septo-Frontal Cell remained intact.

To the best of our knowledge, this is the first case that describes a pneumatized anterior nasal septum continuous with the frontal sinus while a rare anatomical variation, shedding light on the possibility that the condition can lead to potential complications in a patient undergoing nasal surgeries. The role of routine preoperative PNS CT scans in patients undergoing septoplasty is controversial given the adverse effect of radiation and the increased cost [10,11]. However, it can be of important value to evaluate the nasal anatomy and its variations, as in our case, as well as to reduce avoidable surgical failures and complications [12,13].

## Conclusions

This unique case highlights the previously unreported pneumatized anterior nasal septum continuous with the frontal sinus, which we have termed the "Septo-Frontal Cell", and its potential complications that may arise during nasal surgeries. The routine use of preoperative PNS CT scans remains a topic of debate. Our case underscores the potential value of such scans in assessing nasal anatomy and identifying variations to reduce avoidable surgical failures and complications.
